# Differentiation of Low and High‐Grade Meningiomas Using Transfer Learning on MR Images

**DOI:** 10.1049/htl2.70022

**Published:** 2025-10-09

**Authors:** Oktay Fasihi Shirehjini, Farshid Babapour Mofrad, Mohammadreza Shahmohammadi, Fatemeh Karami

**Affiliations:** ^1^ Department of Medical Radiation Engineering SR.C., Islamic Azad University Tehran Iran; ^2^ Functional Neurosurgery Research Center Shohada Tajrish Comprehensive Neurosurgical Center of Excellence Shahid Beheshti University of Medical Sciences Tehran Iran; ^3^ Department of Medical Genetics Applied Biophotonics Research Center SR.C., Islamic Azad University Tehran Iran

**Keywords:** MRI, meningioma, grading, image classification, neural nets, tumours

## Abstract

Despite the advantages of magnetic resonance (MR) images in diagnosing intracranial tumours such as meningiomas, non‐invasive grading remains challenging. In this respect, the potential of transfer learning and data augmentation methods was explored to develop computer‐aided diagnosis (CAD) systems for automatically differentiating between low and high‐grade meningiomas on MR images. Four MR modalities were enrolled, including T1‐weighted, T2‐weighted, fluid‐attenuated inversion recovery and T1‐weighted contrast enhanced and the effectiveness of different data augmentation and deep learning approaches in determining the optimum AI‐based solution for meningioma grading was investigated using a multi‐stage framework, which included image preprocessing, data augmentation by either conventional geometric transformations or fancy principal component analysis (PCA) and the use of ImageNet pre‐trained, well‐known convolutional neural networks (CNNs) for training models with the two widely utilised optimisers of adaptive moment estimation (Adam) and stochastic gradient descent (SGD). Finally, performance was evaluated utilising standard metrics to select the best‐obtained model with optimal approaches. Results indicated that trained visual geometry group (VGG)‐19 with generated images by fancy PCA performed best with an accuracy of 97.80% and 98.90% in classifying unseen samples using SGD and Adam optimisers, respectively. This procedure also yielded acceptably reproducible results in a second study, ensuring the efficiency of each model using a small‐scale dataset. Developing CAD systems using pre‐trained CNNs and fancy PCA is a promising approach for classifying meningiomas on MR images into low and high‐grade categories, which can serve as an alternative to invasive methods and provide valuable assistance for accurate diagnosis before treatment.

## 1 Introduction

Meningiomas are the most widespread primary intracranial tumours, constituting nearly 37% of central nervous system (CNS) neoplasms. These dural‐based tumours arise in the meninges, the protective membranes that surround the brain and spinal cord and originate from arachnoid cap cells that form a morphologically distinct and metabolically active subgroup of arachnoidal cells involved in the resorption of cerebrospinal fluid [1, 2]. According to the World Health Organisation, meningiomas are categorised into low‐grade and high‐grade tumours based on their risk of recurrence and mortality, as determined by tissue analysis and histological features, in which around 80% of meningiomas are low‐grade and 20% are high‐grade [3, 4]. The incidence rate of meningiomas increases with age; thus, these tumours are more prevalent in the elderly population, whereas they are infrequent in children and adolescents. Low‐grade meningiomas are more likely to occur in females, while high‐grade ones are observed more in males [5]. The 10‐year survival rate of cerebral low and high‐grade meningiomas is about 83% and 56%, respectively [6]. Examining acquired brain magnetic resonance (MR) images is the gold standard to detect meningeal tumours, which is a non‐invasive diagnostic method in neuro‐oncology that utilises powerful magnetic fields and radiofrequency pulses to detect mass lesions and other abnormalities and provide images of body organs by rapidly and repeatedly magnetising and relaxing hydrogen molecules [7, 8]. Despite the vital role of MR images in the diagnosis of CNS tumours, pathological examination through tissue biopsy is currently the most reliable grading method, which is not always possible owing to the inaccessibility of the lesion or treatment contraindications and is often associated with severe complications, mostly intracranial haemorrhage and increased post‐biopsy patient mortality [9, 10]. Accurate grading of brain tumours is essential, as it helps to examine the patient's prognosis, the possibility of recurrence and survival and also impacts the determination of the treatment plan [11, 12]. Meningeal tumours have different treatment paradigms depending on their malignancy. For low‐grade meningiomas, surgical intervention is typically beneficial, aiming to achieve complete resection, whereas adjuvant radiotherapy is the standard treatment for high‐grade meningiomas [13].

Artificial intelligence (AI) is the capability of machines to do tasks and make decisions that generally require human intelligence with close or superior performance compared to human experts and professionals [14]. Computer vision (CV) is a field of AI that aims to enable computers to identify specific objects in digitised images [15]. Utilising AI models is one of the most worthwhile plans to develop computer‐aided diagnosis (CAD) systems for high‐priority CV tasks within the healthcare domain, for example, tumour grading, which decreases the need for using invasive methods, can lead to low cost and high accuracy and is a helpful tool to assist specialists in their definitive diagnoses [16, 17]. Instructing intelligent models for classification tasks with desirable results requires deriving the most dominant and non‐redundant features from input samples [18]. Deep learning (DL) is a subfield of machine learning (ML), a branch of AI in which computers can learn from data to carry out requested tasks without being explicitly programmed, that takes advantage of artificial neural networks (ANNs) comprising layers of neurons with trainable parameters for autonomous feature extraction [19, 20]. Convolutional neural networks (CNNs) are ANNs consisting of convolutional layers for automatically extracting features from the images using the convolution operation and fully connected (FC) layers to classify them [21]. Even though DL has performed satisfactorily well in medical image analysis, neural networks are heavily reliant on big data and a large volume of images is required to train the models to avoid overfitting and reach preferable results; however, possessing an extensive number of labelled samples in the medical domain is often challenging due to expensive annotation and limited available data [22, 23]. Transfer learning (TL) and data augmentation are two approaches to compensate for the lack of a large‐scale dataset and its consequences. TL is a method in which formerly learned knowledge of a model in a previous work is utilised to train the models for other assignments. In CNNs, it is feasible to use pre‐trained values for trainable parameters of convolutional layers to extract features from the instances of a new dataset [24]. One of the most favourable datasets containing various images with distinct labels is ImageNet, widely used to provide pre‐trained CNNs [25]. The advantages of fine‐tuning pre‐trained CNNs to train new models include eliminating the need for an enormous labelled dataset to extract and learn ample informational features for an eligible classification, besides reduced training time and computational and memory resources [26]. In recent studies, ImageNet pre‐trained CNNs have been widely used to develop CAD systems for diagnostic purposes, such as diagnosing myocardial infarction and grading glioma tumours using different modalities of MR images [27, 28]. Data augmentation refers to expanding an existing dataset by transforming the original instances, such as medical images, to create new ones with preserved labels. Generating meaningful data helps increase the size of the training data, improve model generalisation and cause accurate classifications by making trained models more robust to unseen data [29]. In the case of image datasets, two standard data augmentation methods are geometric and photometric transformations, including geometric manipulations and colour‐shifting techniques, respectively [30].

**FIGURE 1:**
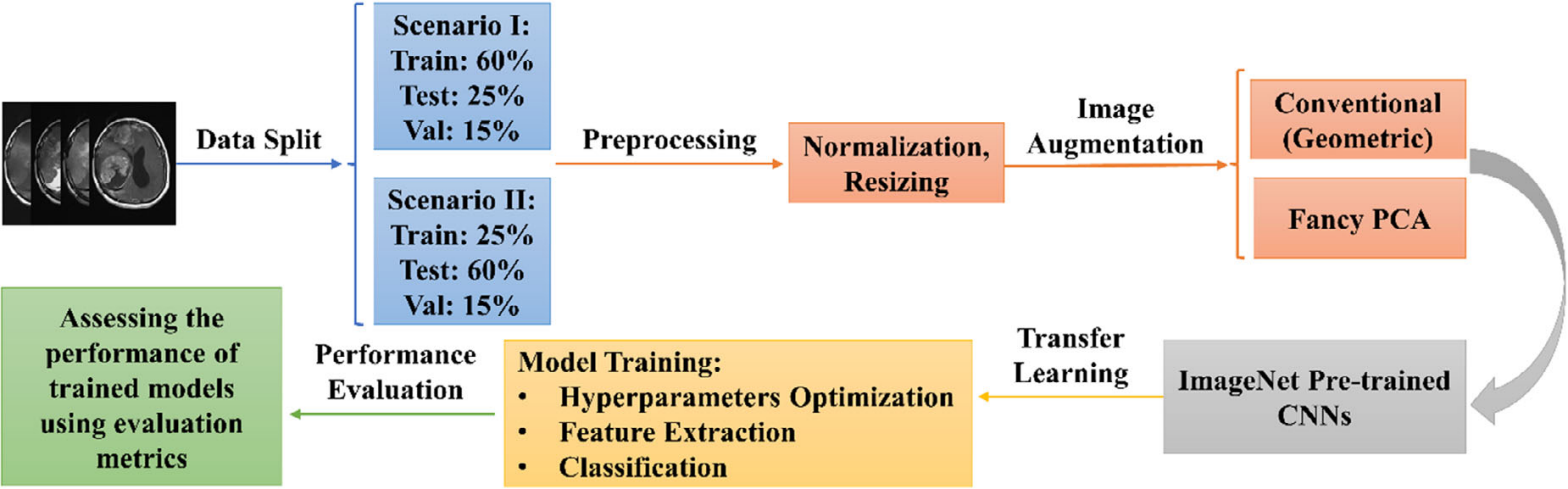
Schematic of the work done during this study.

The main objective of this article was to develop CAD systems to distinguish low‐grade meningiomas from high‐grade ones on four types of MR images, including T1‐weighted, T2‐weighted, fluid‐attenuated inversion recovery (FLAIR) and T1‐weighted contrast enhanced (T1‐CE), taking advantage of ImageNet pre‐trained CNNs, beside two data augmentation methods, that is, geometric transformations (or conventional techniques) and fancy principal component analysis (PCA). The effectiveness of the trained models and their ability to correctly predict the grade of meningiomas on unseen examples for future applications were assessed through classifying test images and computing evaluation metrics. Subsequently, a smaller number of initial images were selected for the training set in a second scenario to evaluate the ability of the considered approach and best‐attained models in achieving noteworthy outcomes with a minimal dataset. Figure 1 illustrates the overall schematic of the research methodology.

To the best of the author's knowledge, the potential of fancy PCA for enhancing training data comprising MR images to identify low and high‐grade meningiomas has not been explored before. In addition, a second study was implemented to ensure the reproducibility of the proposed framework with a limited number of initial images, the capability of generating augmented training sets with informative features and the efficiency of trained models in capturing robust information to achieve desirable results. Identifying an optimal development strategy using the proposed methodologies resulted in a model that minimises dependency on high computational power and large‐scale datasets, which is particularly advantageous for deployment in resource‐constrained clinical environments.

Research contributions are given below:

1. Enrolling MR images of low and high‐grade meningiomas containing T1‐weighted, T2‐weighted, FLAIR and T1‐CE modalities.
2. Implementing two different data augmentation methods of geometric transformations and fancy PCA.
3. Using several well‐known pre‐trained CNNs with different optimisation methods for the development of CAD systems to identify low and high‐grade meningiomas on MR images.
4. Assessing the performance of the models through the computation of evaluation metrics.
5. Training of the models with fewer initial training images in a second study to validate the usefulness of data augmentation methods in generating enough robust features and the efficacy of CNNs in feature extraction and classification with a limited dataset.

The rest of this article is organised as follows. Section 1.1 is the literature review and a summary of related works. Section 2 includes a description of the dataset, image preprocessing and augmentation, model training and methods for performance evaluation. Sections 3 and 4 present achieved results and discuss implemented methods and their outcomes, respectively. Section 5 is the conclusion of this study and proposals for future work.

### 1.1 Related Works

Appointed methods and data in recently published studies about developing models to classify meningiomas into low and high grades using MR images are chronologically reviewed in the following. Yan et al. used texture and shape analysis on a dataset of T1‐weighted, T2‐weighted, FLAIR and T1‐CE images to quantitatively evaluate tumour heterogeneity and morphology. Three classifiers, including logistic regression (LR), naïve Bayes (NB) and support vector machine (SVM), were employed to differentiate low and high‐grade meningiomas, in which using six selected texture and shape features plus SVM resulted in 87% for both accuracy and area under the curve (AUC) scores [31]. Coroller et al. applied radiomic and semantic features to quantify the imaging phenotype using meningioma T1‐CE images and an AUC of 86% was obtained utilising random forest (RF) to classify images into low and high grades after combining both groups of features [32]. Laukamp et al. extracted shape and texture features from T1‐weighted, T2‐weighted, T1‐CE, FLAIR, diffusion weighted imaging (DWI) and apparent diffusion coefficient (ADC) maps after tumour segmentation and reached an AUC score of 91% using a multivariate LR model [33]. Hamerla et al. extracted radiomic features from ADC maps, T1‐weighted, T2‐weighted, FLAIR and T1‐CE images of meningiomas after tumour segmentation to classify them into low and high grades using RF, SVM, extreme gradient boosting (XGBoost) and multilayer perceptron. The best AUC was 97%, calculated for the trained model with 16 out of 10914 derived radiomic features and XGBoost [34]. Ke et al. extracted texture features from pre‐processed T1‐weighted, T2‐weighted and T1‐CE images of low and high‐grade meningiomas, combined them with radiological characteristics and utilised a two‐step feature selection to reduce combined features. The classification was performed using the SVM algorithm, yielding 89% accuracy and 91% AUC for the validation cohort [35]. Chu et al. extracted texture features from the lesions in recruited enhanced T1‐weighted images of cases with low and high‐grade meningiomas. Least absolute shrinkage and selection operator (LASSO) and LR methods were employed for dimension reduction and classification, respectively and the model reached an accuracy and AUC of 92.9% and 94.8%, respectively [36]. Wodzinski et al. segmented tumours on T1‐weighted post‐contrast images of low and high‐grade meningiomas and after spatial image normalisation and data augmentation based on colour and affine transformations, pre‐processed cases were passed to a trained CNN that yielded an accuracy of 74% [37]. Hu et al. recruited different types of MR images and extracted radiomic features from segmented tumours. The performance of radiomic models was evaluated by a nested leave‐one‐out cross‐validation (LOOCV) approach combining LASSO and RF that was trained without subsampling or with the synthetic minority over‐sampling technique (SMOTE). The trained model, using T1‐weighted and T2‐weighted images, susceptibility weighted imaging (SWI) and ADC maps, achieved the best AUC scores of 84% and 81% without and with subsampling, respectively [38]. Han et al. had T1 FLAIR, CE‐T1 FLAIR and T2‐weighted images of low and high‐grade meningiomas. Radiomic feature analysis was done by removing features with low variance, univariate feature selection and LASSO to train the models using six different classifiers, including LR, SVM, K‐nearest neighbours, decision tree and RF. SVM obtained the best model with an AUC of 95.6% [39]. Yang et al. developed models to differentiate low and high‐grade meningiomas on T1‐weighted images using extracted radiomics and DL features from axial and sagittal images and the maximum slice of the axial tumour lesion, respectively and SMOTE to balance the sample numbers. They selected optimal discriminative features for model building and the LightGBM algorithm to implement classification by combining radiomic and DL features, which achieved 92.6% accuracy and an AUC score of 93.5% [40]. Duan et al. extracted texture features from the region of interest (ROI) of enhanced T1‐weighted images of low and high‐grade meningiomas to classify them using different ML algorithms. The highest accuracy for radiomic models was attained using the SVM classifier, with accuracy and AUC of 79% and 88.4%, respectively [41]. Vassantachart et al. collected T1‐CE and FLAIR images of cases with low and high‐grade meningiomas. They used an asymmetric CNN architecture constructed with two encoding paths with the same size kernel and different filters to weigh the spatial features of each sequence separately, in addition to tenfold cross‐validation to appraise model performance and achieve an accuracy of 90% [42]. Chen et al. conducted a retrospective multicentre study using T1‐weighted, T2‐weighted and T1‐CE images of low and high‐grade meningiomas. Agreement between radiomic features from manual and automatic segmentations employing a modified attention U‐Net was assessed using the intraclass correlation coefficient (ICC). After univariate and minimum‐redundancy‐maximum‐relevance feature selection, L1‐regularised LR was utilised to classify the images and AUC scores of 88% and 91% were achieved utilising automatic and manual segmentation, respectively [43]. Duan et al. extracted radiomic features from ROIs on enhanced T1‐weighted images of low and high‐grade meningiomas and the Spearman correlation analysis and LASSO regression were used to find the valuable features. The predictive models were constructed by NB, gradient boosting decision tree and SVM, in which the model with the NB classifier had the highest AUC of 77.3% [44]. Jun et al. collected a dataset including T2‐weighted and T1‐CE images of patients with low and high‐grade meningiomas and constructed a two‐stage DL grading model based on U‐net and residual network (ResNet) architectures for segmentation and classification, which had the best accuracy of 72.1% and AUC of 77% using both types of images [45]. Adil et al. applied topologic and geometric feature analysis after implementing segmentation to predict the grade of meningioma tumours on skull‐based FLAIR images using the XGBoost classification method and achieved an AUC of 75% for the trained ML model [46].

## 2 Materials and Methods

### 2.1 Dataset

**FIGURE 2:**
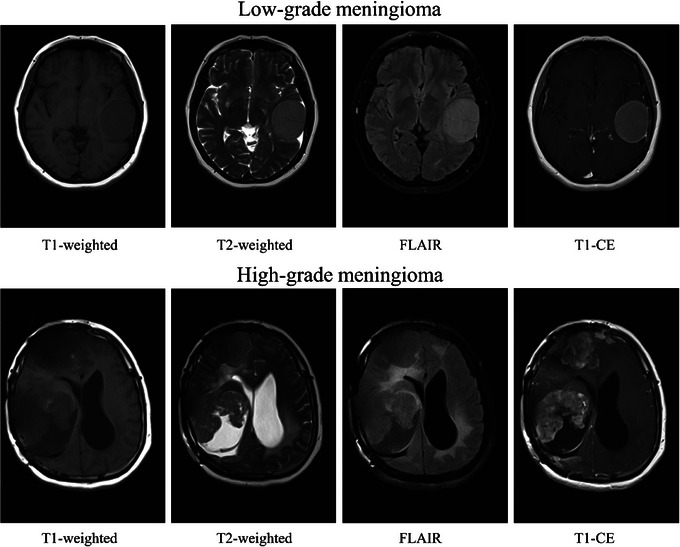
Samples of MR images with low and high‐grade meningiomas.

The dataset contained four modalities of brain axial MR images, including T1‐weighted, T2‐weighted, FLAIR and T1‐CE, belonging to 19 and 10 patients with pathologically confirmed low and high‐grade meningiomas, respectively, acquired by a 1.5 Tesla MR scanner in tagged image file format, the preferable format when high‐quality images are required, such as medical diagnosis, since it is versatile, supports the full range of image sizes, resolutions and colour depths and retains the original image quality [47]. Table 1 depicts the number of male and female patients, their age range and average age for each grade. Table 2 presents the number of images categorised by modality and label separately. Figure 2 displays random T1‐weighted, T2‐weighted, FLAIR and T1‐CE images from the dataset with low and high‐grade meningiomas.

**TABLE 1:** Number, age range and average age of the patients.

Grade	Female (#)	Male (#)	Min. age	Max. age	Mean (std)
Low‐grade	14	5	32	73	52.78(12.03)
High‐grade	5	5	26	77	46.3(14.86)
Total	19	10	26	77	50.55(13.18)

*Note*: std, standard deviation.

**TABLE 2:** Number of initial images.

	Modality			
Grade	T1‐weighted	T2‐weighted	FLAIR	T1‐CE
Low‐grade	103	115	92	53
High‐grade	51	66	64	55

### 2.2 Preprocessing

In the first preprocessing step, a hold‐out split strategy was utilised to divide the initial images into distinct training, test and validation groups, in which 60% of the images were randomly assigned to the training set and 25% and 15% of the remaining images were devoted to test and validation sets, respectively [48]. This data splitting approach allocates a comparatively large number of images to the test set to ensure a more rigorous and robust evaluation of the models’ generalisation performance on unseen data, a widely adopted and efficient approach in medical image analysis studies [49, 50, 51]. Afterwards, image augmentation was done using the photometric transformation method of fancy PCA or conventional geometric transformation techniques, including horizontal flipping, rotating between zero and five degrees and width or height shifting up to 10% of the image size, in which the number of training images utilising each method was increased to 500 for both low and high grades, respectively. In the last step of preparing the images for training, all of them were resized to 224 × 224 after zero padding and the intensity values of their pixels were normalised by transforming them to the range between zero and one before importing images to each CNN, according to [52].

#### 2.2.1 Fancy PCA

**FIGURE 3:**
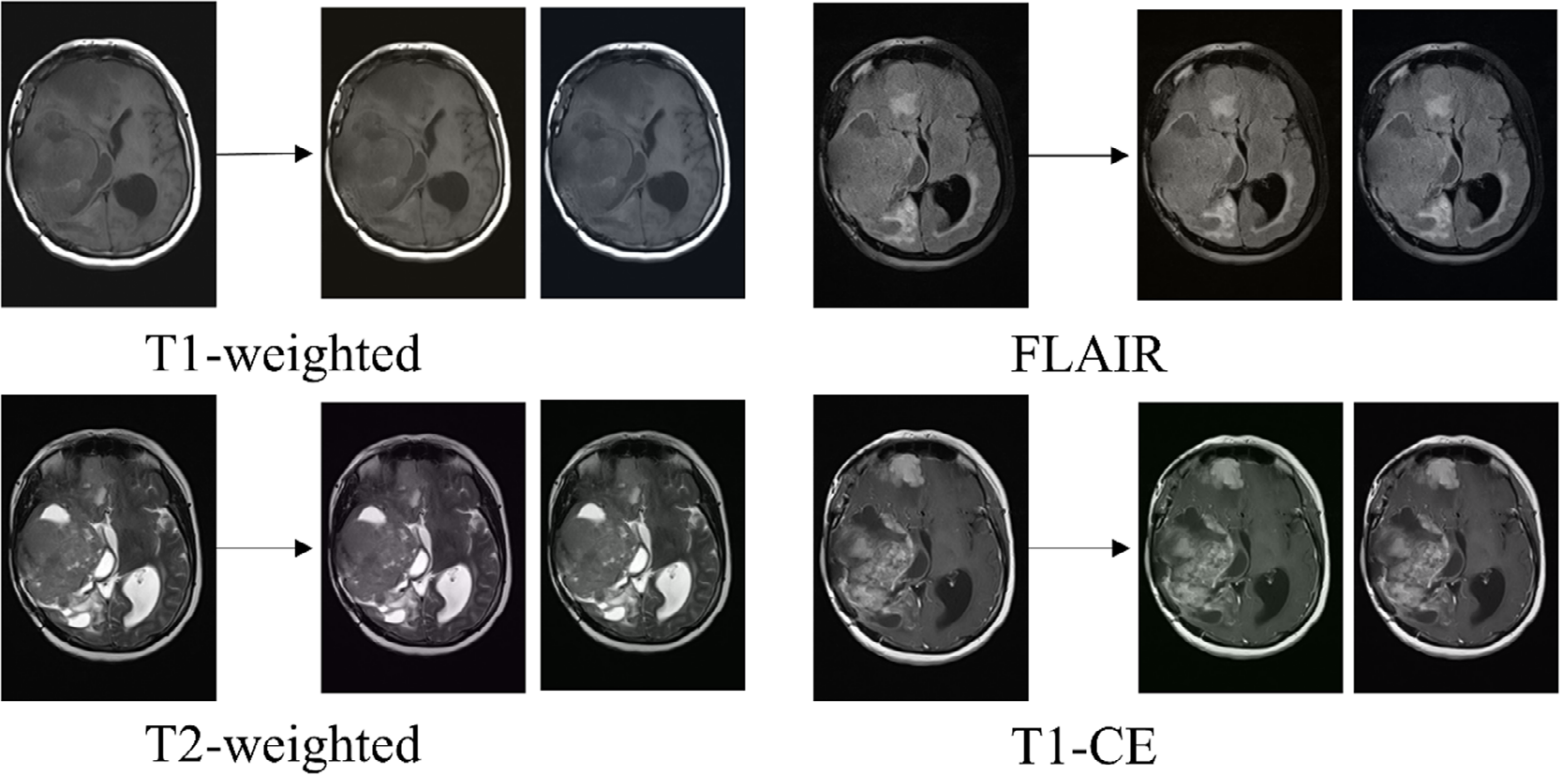
Samples of meningioma MR images and two artificially generated images with fancy PCA (images on the left and right of the arrows are original and augmented images, respectively).

Fancy PCA, or PCA colour augmentation, is a photometric image augmentation method in which PCA is performed on the set of pixel values and multiples of the identified principal components are added to each image, with magnitudes proportional to corresponding eigenvalues times a random variable drawn from a Gaussian with mean zero and standard deviation 0.1 [53]. Fancy PCA has been successfully utilised to expand the training set in various medical image analysis tasks, such as breast cancer diagnosis on mammograms, ultrasound images or histopathology images, detecting cancer in mass spectrometry imaging (MSI) data and diabetic foot identification on plantar thermograms [54, 55, 56]. Equation (1) represents the intensity value of each pixel with three channels at spatial location (*x,y*) and Equation (2) is the added quantity where *p_i_* and *λ_i_* are the *i*th eigenvectors and eigenvalues of the 3 × 3 covariance matrix of pixel values, respectively and *α_i_* is the randomly drawn variable once per image that scales the contribution of each principal component [53]. Figure 3 shows four random initial MR images with different modalities and two generated images by fancy PCA for each.

(1) $$I_{xy}={\left[I_{xy}^{R},I_{xy}^{G},I_{xy}^{B}\right]}^{T}\;$$

(2) $$\;\Delta I_{xy}=\left[p_{1},p_{2},p_{3}\right]\;{\left[\alpha_{1}\lambda_{1},\alpha_{2}\lambda_{2},\alpha_{3}\lambda_{3}\right]}^{T}$$

### 2.3 Training of the Models

After the preprocessing section, 15 well‐known ImageNet pre‐trained CNNs that have previously performed well in medical image processing, described in Sections 2.3.1–2.3.8, were employed for developing CAD systems to discern low and high‐grade meningioma tumours on MR images [57, 58, 59]. For this purpose, synthetically made images by conventional techniques or fancy PCA were imported to each network to classify them through updating trainable parameters in FC layers using automatically extracted features in previous layers. Optimisation methods to train the models were adaptive moment estimation (Adam) and stochastic gradient descent (SGD) with Nesterov acceleration, where β1 and β2, or the decay rates of Adam, were 0.9 and 0.99, respectively; the momentum value of SGD was 0.99 and both optimisers had a learning rate of 0.001 [60]. The number of epochs and batch size to train the models were 50 and 32, respectively. All models used a binary cross‐entropy cost function for computing training errors and a sigmoid function to implement binary classifications in the top FC layer [61, 62]. The optimal hyperparameters were selected after testing various commonly used values in research endeavours, resulting in strong performance consistent with previous studies on classification models for medical images [51, 63].

#### 2.3.1 NASNet

Neural architecture search network, or NASNet, models look for the ideal CNN construct as a reinforcement learning problem and have two types of convolutional cells, named normal and reduction, that return feature maps with the same dimension and reduced‐size feature maps by a factor of two, respectively, searched by a controller recurrent neural network. NASNet Mobile and NASNet Large were NASNet variants in this study, which differ in size and number of parameters [64].

#### 2.3.2 ResNetv2

ResNetV2 models are modified versions of ResNets, which are known for utilising skip connections to perform residual learning, thereby preventing accuracy degradation problems that occur when network depth increases. Modifications are made in the propagation formulation of the connections between blocks, where batch normalisation is done before each weight layer, which improves generalisation. ResNet50‐V2, ResNet101‐V2 and ResNet152‐V2 with 50, 101 and 152 layers with trainable parameters, respectively, were employed variants of ResNetv2 in this research [65].

#### 2.3.3 Xception

Xception networks contain the entry, middle, repeated eight times and exit flows consisting of inception modules with depth‐wise separable convolutional layers, comprising a 3 × 3 convolutional layer with one kernel and a 1 × 1 convolutional layer with a particular number of kernels and residual connections [66].

#### 2.3.4 Inception‐v3

Inception‐V3 comprises several symmetric and asymmetric building blocks, including two structurally different reduction blocks and three disparate designs of inception modules with sequential convolutional and pooling layers placed in parallel [67].

#### 2.3.5 Inception‐ResNet‐v2

Inception‐ResNet‐v2 is made up of modifying the structure of Inception‐V4 by changing inception blocks with inception‐ResNet blocks in which four inception‐A, seven inception‐B and three inception‐C blocks are replaced with five inception‐ResNet‐A, ten inception‐ResNet‐B and five inception‐ResNet‐C blocks, respectively [68].

#### 2.3.6 DenseNet

DenseNet‐121, DenseNet‐169 and DenseNet‐201 are three variants of dense networks with 121, 169 and 201 layers with trainable parameters, respectively, used in this study. These networks have four dense blocks comprising consecutive pairs of 1 × 1 and 3 × 3 convolutional kernels and three transition layers containing a 1 × 1 convolutional layer before a 2 × 2 average pooling layer with a stride of two [69].

#### 2.3.7 MobileNet

MobileNets are lightweight CNNs that use depth‐wise separable convolutional layers to reduce the size and number of trainable parameters compared to standard convolutional layers. MobileNetV2 is a variant of the MobileNet models that leverages bottleneck blocks [70, 71].

#### 2.3.8 VGGNet

These networks consist of five blocks, each comprising a different number of 3 × 3 convolutional kernels, followed by a 2 × 2 maximum pooling layer with a stride of two. VGG‐16 and VGG‐19 were visual geometry group (VGG) models in this study, consisting of 16 and 19 layers with trainable parameters, respectively [72].

### 2.4 Performance Evaluation

The performance of models is assessed by classifying test images and reporting computed values for multiple evaluation metrics, that is, accuracy, precision, sensitivity and F‐score, based on the classification results [73]. Equations (3)–(6) are mentioned metrics in which true positive (TP) and false negative (FN) are images with low‐grade meningiomas that the models predicted correctly and incorrectly, respectively and true negative (TN) and false positive (FP) are images with high‐grade meningiomas that the models predicted correctly and incorrectly, respectively. AUC scores for receiver operating characteristic (ROC) curves of each trained model, shown in Equation (7), were also computed, where *S*_p_ denotes the sum of all positive samples ranked and *n*_p_ and *n*_n_ are the number of positive and negative samples, respectively [74].

(3) $$\mathrm{Accuracy}\;=\frac{\mathrm{TP}\;+\;\mathrm{TN}}{\mathrm{TP}\;+\;\mathrm{TN}\;+\;\mathrm{FP}\;+\;\mathrm{FN}}\;$$

(4) $$\mathrm{Precision}\;=\frac{\mathrm{TP}}{\mathrm{TP}\;+\;\mathrm{FP}}$$

(5) $$\mathrm{Sensitivity}\;=\frac{\mathrm{TP}}{\mathrm{TP}\;+\;\mathrm{FN}}$$

(6) $$F-\mathrm{score}=\frac{2\left(\mathrm{Sensitivity}\;\times \;\mathrm{Precision}\right)}{\mathrm{Sensitivity}\;+\;\mathrm{Precision}}\;$$

(7) $$\mathrm{AUC}=\frac{S_{p}-n_{p}\left(n_{n}+1\right)/2}{n_{p}n_{n}}\;$$

## 3 Results

**FIGURE 4:**
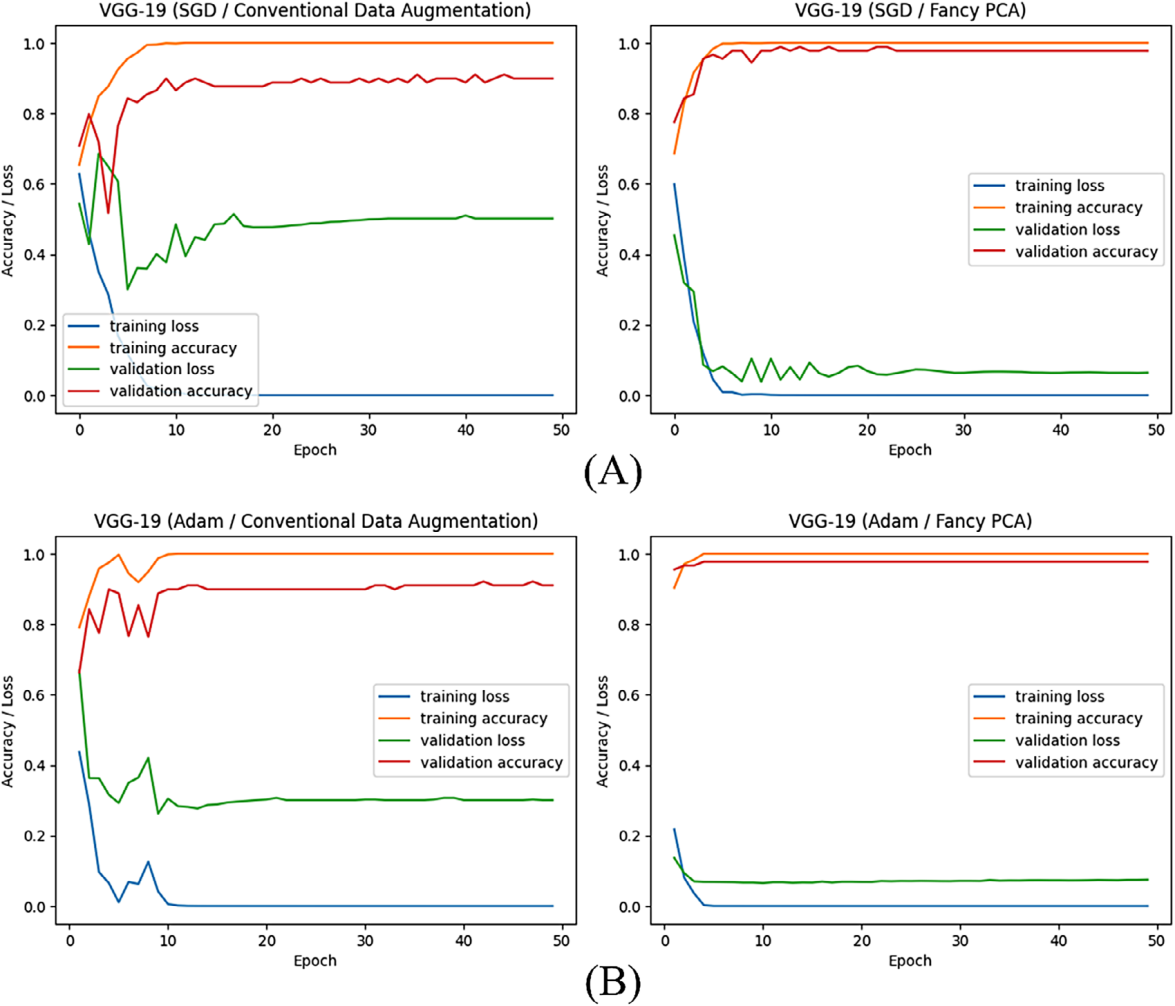
(A) and (B) are the accuracy and loss values of training and validation samples during the training process of VGG‐19 trained with created images by conventional techniques or fancy PCA using SGD and Adam optimisers, respectively.

**FIGURE 5:**
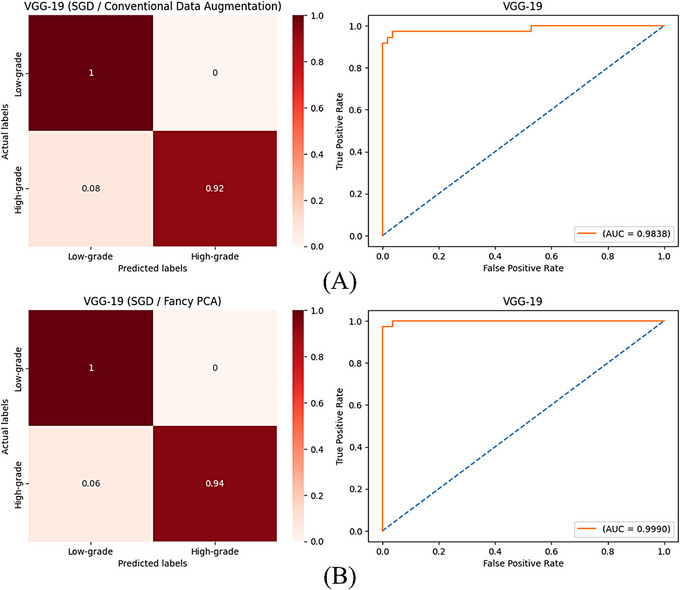
(A) and (B) are confusion matrices with normalised values and ROC curves of VGG‐19 plus SGD optimiser trained with augmented images by conventional techniques and fancy PCA, respectively.

**FIGURE 6:**
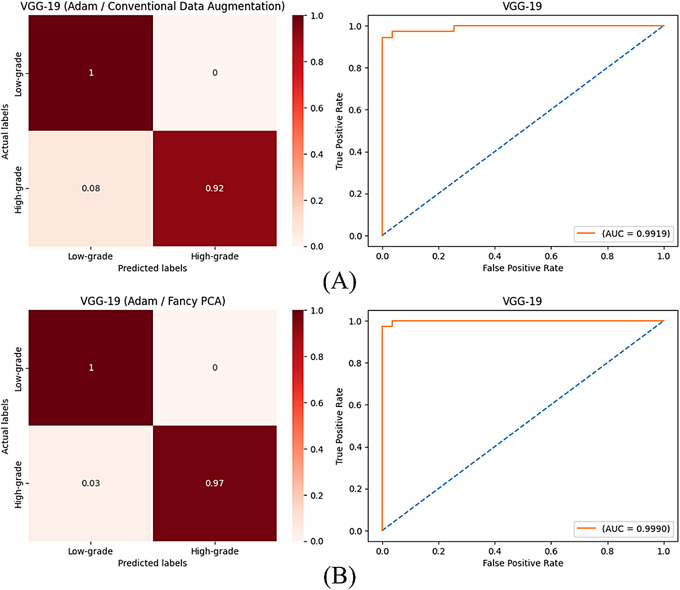
(A) and (B) are confusion matrices with normalised values and ROC curves of VGG‐19 plus Adam optimiser trained with augmented images by conventional techniques and fancy PCA, respectively.

Images of the test set were classified by trained models to compute evaluation metrics and appraise their performance. Tables 3 and 4 are classification results for the trained models with artificially made images by conventional techniques and fancy PCA, respectively. Figure 4 shows computed accuracy and loss values after classifying training and validation images at each epoch during the training process of VGG‐19 with different augmented training images and optimisers. Figures 5 and 6 show confusion matrices with normalised values and ROC curves for trained VGG‐19 with SGD and Adam optimisers, respectively, using generated images by conventional techniques or fancy PCA.

**TABLE 3:** Classification results for the trained models with augmented images by conventional techniques.

		Adam					SGD				
No.	Feature extractor	Accuracy	Precision	Sensitivity	F‐score	AUC	Accuracy	Precision	Sensitivity	F‐score	AUC
1	NasNet Mobile	78.02%	68.18%	83.33%	75.00%	83.28%	81.31%	73.17%	83.33%	77.92%	85.85%
2	NasNet Large	79.12%	77.41%	66.66%	71.64%	87.97%	78.02%	78.57%	61.11%	68.75%	86.21%
3	Xception	76.92%	66.66%	83.33%	74.07%	86.96%	78.02%	70.00%	77.77%	73.68%	88.23%
4	Inception‐V3	81.31%	95.23%	55.55%	70.17%	89.79%	79.12%	68.08%	88.88%	77.10%	87.02%
5	Inception‐ResNet‐v2	82.41%	85.71%	66.66%	75.00%	91.31%	86.81%	78.57%	**91.66%**	84.61%	91.41%
6	ResNet50‐V2	78.02%	73.52%	69.44%	71.42%	83.53%	74.72%	68.57%	66.66%	67.60%	83.23%
7	ResNet101‐V2	85.71%	84.84%	77.77%	81.15%	91.66%	84.61%	80.55%	80.55%	80.55%	89.39%
8	ResNet‐152‐V2	81.31%	77.14%	75.00%	76.05%	87.72%	79.12%	71.79%	77.77%	74.66%	86.81%
9	DenseNet‐121	80.21%	70.45%	86.11%	77.50%	88.38%	83.51%	78.37%	80.55%	79.45%	85.85%
10	DenseNet‐169	78.02%	73.52%	69.44%	71.42%	89.59%	75.82%	70.58%	66.66%	68.57%	90.90%
11	DenseNet‐201	90.10%	81.39%	97.22%	88.60%	93.48%	82.41%	72.72%	88.88%	80.00%	92.47%
12	MobileNet	93.40%	96.87%	86.11%	91.17%	97.32%	90.10%	93.54%	80.55%	86.56%	92.97%
13	MobileNetV2	84.61%	82.35%	77.77%	80.00%	90.80%	85.71%	81.08%	83.33%	82.19%	91.66%
14	VGG‐16	93.40%	94.11%	88.88%	91.42%	98.08%	90.10%	93.54%	80.55%	86.56%	98.18%
15	VGG‐19	**96.70%**	**100.00%**	**91.66%**	**95.65%**	**99.19%**	**96.70%**	**100.00%**	**91.66%**	**95.65%**	**98.38%**

*Note*: Bold numbers are the most elevated values.

**TABLE 4:** Classification results for the trained models with augmented images by fancy PCA.

		Adam					SGD				
No.	Feature extractor	Accuracy	Precision	Sensitivity	F‐score	AUC	Accuracy	Precision	Sensitivity	F‐score	AUC
1	NasNet Mobile	79.12%	70.73%	80.55%	75.32%	86.71%	83.51%	80.00%	77.77%	78.87%	88.23%
2	NasNet Large	83.51%	80.00%	77.77%	78.87%	91.51%	83.51%	78.37%	80.55%	79.45%	90.55%
3	Xception	78.02%	75.00%	66.66%	70.58%	88.43%	81.31%	75.67%	77.77%	76.71%	89.74%
4	Inception‐V3	84.61%	80.55%	80.55%	80.55%	92.82%	81.31%	70.21%	91.66%	79.51%	91.56%
5	Inception‐ResNet‐v2	83.51%	88.88%	66.66%	76.19%	93.13%	85.71%	81.08%	83.33%	82.19%	92.57%
6	ResNet50‐V2	76.92%	77.77%	58.33%	66.66%	86.21%	72.52%	70.37%	52.77%	60.31%	84.69%
7	ResNet101‐V2	93.40%	96.87%	86.11%	91.17%	94.89%	84.61%	82.35%	77.77%	80.00%	92.32%
8	ResNet‐152‐V2	85.71%	84.84%	77.77%	81.15%	89.79%	83.51%	80.00%	77.77%	78.87%	90.85%
9	DenseNet‐121	85.71%	82.85%	80.55%	81.69%	91.41%	85.71%	82.85%	80.55%	81.69%	89.89%
10	DenseNet‐169	87.91%	87.87%	80.55%	84.05%	94.14%	86.81%	83.33%	83.33%	83.33%	93.78%
11	DenseNet‐201	93.40%	87.50%	**97.22%**	92.10%	97.17%	89.01%	88.23%	83.33%	85.71%	96.51%
12	MobileNet	92.30%	93.93%	86.11%	89.85%	98.03%	90.10%	90.90%	83.33%	86.95%	93.58%
13	MobileNetV2	84.61%	84.37%	75.00%	79.41%	95.00%	86.81%	83.33%	83.33%	83.33%	96.16%
14	VGG‐16	96.70%	**100.00%**	91.66%	95.65%	99.79%	**97.80%**	**100.00%**	**94.44%**	**97.14%**	99.69%
15	VGG‐19	**98.90%**	**100.00%**	**97.22%**	**98.59%**	**99.90%**	**97.80%**	**100.00%**	**94.44%**	**97.14%**	**99.90%**

*Note*: Bold numbers are the most elevated values.

### 3.1 The Second Scenario

**FIGURE 7:**
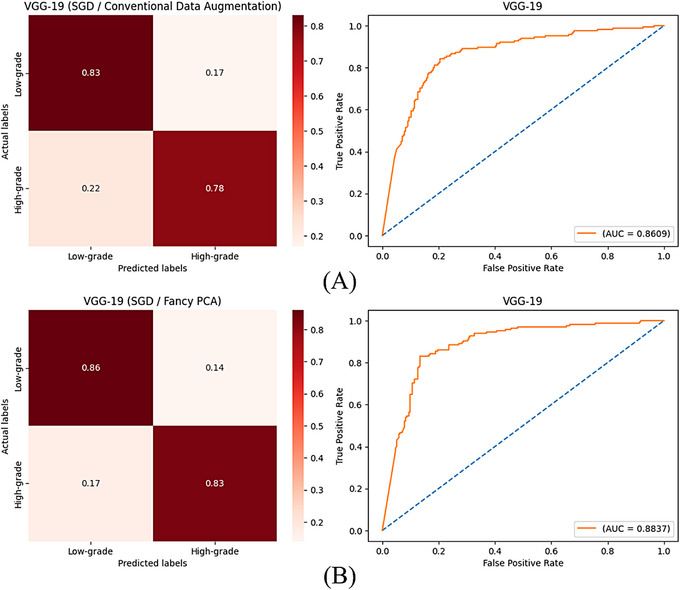
(A) and (B) are confusion matrices with normalised values and ROC curves of VGG‐19 plus SGD optimiser trained with augmented images by conventional techniques and fancy PCA in the second scenario, respectively.

**FIGURE 8:**
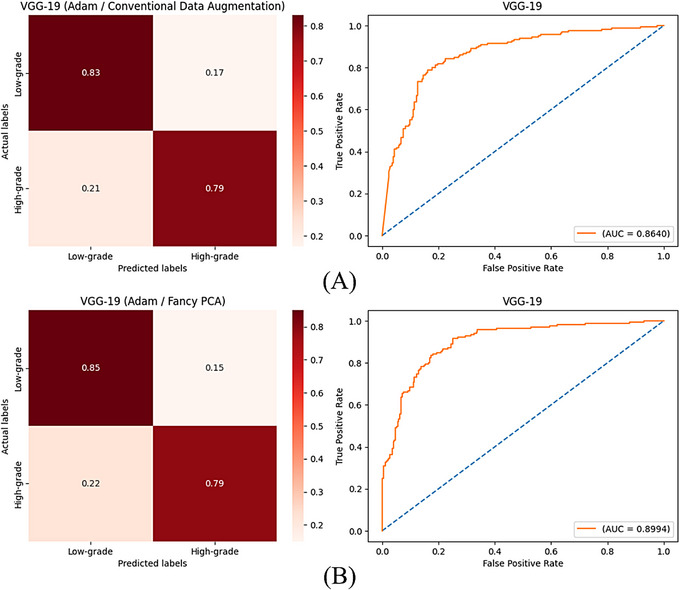
(A) and (B) are confusion matrices with normalised values and ROC curves of VGG‐19 plus Adam optimiser trained with augmented images by conventional techniques and fancy PCA in the second, respectively.

In a second scenario, contrary to the usual study, 60% and 25% of the images were selected for the test and training sets, respectively and 15% of the images were used as the validation group, using the hold‐out split method. All other steps, such as data augmentation methods and the training process, were accomplished as before. This scenario was done to appraise the performance of CNNs in being proficient at fulfilling a well‐qualified feature extraction to reach properly approved classifications with a limited dataset and evaluate the efficiency of data augmentation methods in generating synthetic images with sufficient informative features to train models with satisfactory results while having a small number of initial training images. Tables 5 and 6 show values of computed evaluation metrics for the trained models with augmented images by conventional techniques and fancy PCA in this scenario, respectively and Figures 7 and 8 show confusion matrices with normalised values and ROC curves of VGG‐19 networks trained with generated images by conventional techniques or fancy PCA using SGD and Adam optimisers, respectively.

**TABLE 5:** Classification results for the trained models with augmented images by conventional techniques (the second scenario).

		Adam					SGD				
No.	Feature extractor	Accuracy	Precision	Sensitivity	F‐score	AUC	Accuracy	Precision	Sensitivity	F‐score	AUC
1	NasNet Mobile	67.78%	59.61%	56.36%	57.94%	72.58%	69.21%	66.07%	44.84%	53.42%	72.28%
2	NasNet Large	66.82%	57.83%	58.18%	58.00%	70.05%	69.45%	63.50%	52.72%	57.61%	72.56%
3	Xception	72.79%	64.73%	67.87%	66.27%	78.37%	74.70%	70.34%	61.81%	65.80%	77.27%
4	Inception‐V3	68.49%	72.00%	32.72%	45.00%	70.31%	68.73%	61.48%	55.15%	58.14%	71.95%
5	Inception‐ResNet‐v2	70.16%	60.30%	70.90%	65.18%	75.64%	70.16%	62.65%	60.00%	61.30%	75.62%
6	ResNet50‐V2	71.59%	66.42%	56.36%	60.98%	73.71%	68.97%	61.14%	58.18%	59.62%	70.45%
7	ResNet101‐V2	70.16%	64.08%	55.15%	59.28%	75.98%	72.31%	68.42%	55.15%	61.07%	77.16%
8	ResNet‐152‐V2	71.83%	65.98%	58.78%	62.17%	77.16%	68.01%	59.74%	57.57%	58.64%	72.81%
9	DenseNet‐121	70.64%	62.65%	63.03%	62.83%	75.96%	72.31%	63.38%	70.30%	66.66%	78.32%
10	DenseNet‐169	75.17%	70.46%	63.63%	66.87%	78.46%	73.50%	64.51%	72.72%	68.37%	79.88%
11	DenseNet‐201	73.50%	66.26%	66.66%	66.46%	78.34%	71.12%	62.79%	65.45%	64.09%	76.39%
12	MobileNet	77.32%	75.00%	63.63%	68.85%	81.29%	75.65%	72.34%	61.81%	66.66%	80.50%
13	MobileNetV2	69.21%	62.00%	56.36%	59.04%	75.08%	73.50%	71.09%	55.15%	62.11%	76.47%
14	VGG‐16	80.66	73.86	**78.78%**	76.24%	**88.74%**	80.66%	72.58%	**81.81%**	**76.92%**	**88.31%**
15	VGG‐19	**81.14%**	**74.71%**	**78.78%**	**76.69%**	86.40%	**81.14%**	**75.29%**	77.57%	76.41%	86.09%

*Note*: Bold numbers are the most elevated values.

**TABLE 6:** Classification results for the trained models with augmented images by fancy PCA (the second scenario).

		Adam					SGD				
No.	Feature extractor	Accuracy	Precision	Sensitivity	F‐score	AUC	Accuracy	Precision	Sensitivity	F‐score	AUC
1	NasNet Mobile	67.30%	58.86%	56.36%	57.58%	72.07%	69.68%	61.87%	60.00%	60.92%	72.29%
2	NasNet Large	66.34%	57.50%	55.75%	56.61%	69.60%	65.15%	55.75%	55.75%	55.75%	69.01%
3	Xception	72.55%	64.20%	68.48%	66.27%	78.34%	72.07%	64.28%	65.45%	64.86%	77.75%
4	Inception‐V3	69.68%	65.32%	49.09%	56.05%	71.99%	69.45%	64.12%	50.90%	56.75%	71.79%
5	Inception‐ResNet‐v2	71.12%	61.95%	69.09%	65.32%	75.91%	71.36%	62.29%	69.09%	65.51%	76.16%
6	ResNet50‐V2	72.31%	68.14%	55.75%	61.33%	74.62%	67.54%	60.13%	52.12%	55.84%	70.57%
7	ResNet101‐V2	70.16%	64.28%	54.54%	59.01%	75.87%	67.78%	61.19%	49.69%	54.84%	75.98%
8	ResNet‐152‐V2	73.74%	68.21%	62.42%	65.18%	76.97%	71.59%	65.75%	58.18%	61.73%	76.73%
9	DenseNet‐121	72.31%	65.60%	62.42%	63.97%	77.13%	71.36%	63.80%	63.03%	63.41%	74.92%
10	DenseNet‐169	73.50%	68.49%	60.60%	64.30%	76.95%	71.59%	64.19%	63.03%	63.60%	76.29%
11	DenseNet‐201	73.03%	65.85%	65.45%	65.65%	78.01%	71.12%	63.41%	63.03%	63.22%	77.16%
12	MobileNet	77.80%	75.35%	64.84%	69.70%	82.02%	77.32%	74.30%	64.84%	69.25%	81.56%
13	MobileNetV2	70.40%	63.57%	58.18%	60.75%	75.38%	69.68%	63.19%	55.15%	58.89%	75.10%
14	VGG‐16	81.38%	76.36%	76.36%	76.36%	**90.28%**	84.48%	77.77%	**84.84%**	**81.15%**	**90.09%**
15	VGG‐19	**82.57%**	**77.71%**	**78.18%**	**77.94%**	89.94%	**84.72%**	**79.19%**	83.03%	81.06%	88.37%

*Note*: Bold numbers are the most elevated values.

## 4 Discussion

This study aimed to develop a practical approach for non‐invasively distinguishing between low and high‐grade meningiomas using MR images with different modalities. In this respect, in addition to implementing two data augmentation methods, that is, conventional geometric transformations and fancy PCA, to increase the number of training images for DL tasks, fifteen well‐known CNNs, already trained on the ImageNet dataset, alongside Adam and SGD optimisers with particular hyperparameters, were used to derive features from the images automatically and classify them. Afterwards, to find the best‐trained model, the performance of each one was appraised by computing criterion evaluation metrics after predicting the grade of meningiomas on MR images of the test set. Investigating achieved results showed that various methods and procedures for data augmentation and model training resulted in different outcomes, as explained in the following.

According to the classification results, in both scenarios, VGG‐19 could perform best among all trained models with different optimisation methods and training sets and VGG‐16 ranked second with slightly similar performance. In the first scenario, VGG‐19 achieved an accuracy of 96.70% using both Adam and SGD optimisers when it was trained with created images by conventional techniques; however, despite computing the same values for precision, sensitivity and F‐score along with accuracy, training of this model with Adam yielded a better AUC score of 99.19%, compared to 98.38% that was obtained when it was trained with SGD. Using augmented training images by fancy PCA resulted in the best accuracy of 98.90% and 97.80% for VGG‐19 plus Adam and SGD optimisers, respectively and the first one achieved the best performance among all trained models in this research. Trained VGG‐16 using SGD and generated images by fancy PCA, also obtained an accuracy of 97.80% and equal values with the VGG‐19 plus SGD model for all evaluation metrics except AUC, which was 99.69% compared to 99.90% that was attained for the model containing VGG‐19. Comparing the plotted curves of computed accuracies and losses for training and validation samples at each epoch during the training of VGG‐19 with different training sets, displayed in Figure 4, indicates that using synthetically made images by fancy PCA rather than conventional techniques to train the best‐obtained models yielded significantly higher and lower accuracy and loss values, respectively and more stable and unchanging curves for both training and validation sets after a certain number of epochs to avoid over‐fitting. Calculated confusion matrices and plotted ROC curves for trained VGG‐19 networks, illustrated in Figures 5 and 6, exhibit that these models could successfully detect all low‐grade meningiomas on the test set regardless of optimiser or training set, but using generated training images by fancy PCA instead of conventional techniques improved the classification accuracy of high‐grade meningiomas from 92% to 94% and 97% taking advantage of SGD and Adam optimisers, respectively and slightly increased AUC values as well. The results show the potency of fancy PCA for data augmentation and its influential ability to create synthetic images comprising instructive features from initial images for well‐fitted and proper classifications.

In the second scenario, training of VGG‐19 utilising augmented images by conventional techniques had the best accuracy of 81.14% for both Adam and SGD optimisers between all models; nevertheless, AUC values for trained models with Adam and SGD were 86.40% and 86.09%, respectively and VGG‐16 obtained the best AUC values in this part which were 88.74% and 88.31% using Adam and SGD, respectively. When the models were trained with images created by fancy PCA, VGG‐19 achieved the best accuracy with both optimisation methods again, which were 82.57% and 84.72% using Adam and SGD, respectively. A comparison of confusion matrices and ROC curves of trained VGG‐19 networks in this scenario, represented in Figures 7 and 8, shows that training of the models with both Adam and SGD optimisers using augmented images by fancy PCA rather than conventional techniques caused more accurate classification of low and high‐grade meningiomas of the test set and increased AUC values. Comparing results deduces the reproducibility of the employed method and VGG‐19 could reach the highest accuracies and acceptable results in the second scenario, as well as the usual study, where training of the models with Adam and fancy PCA instead of SGD and geometric transformations, respectively, yielded a better performance. Perusing the best‐obtained models in the second scenario inferred the notable robustness of VGG‐19 in extracting efficacious features from the images for desired classifications, even with a small initial training set and the effectiveness of the fancy PCA data augmentation method in generating synthetic images containing adequate informative features using a restricted number of images.

Furthermore, to statistically validate the performance improvement, a paired *t*‐test was conducted between models with the same optimiser but using different data augmentation methods. Evaluation was based on classification accuracy and AUC, as widely recognised, interpretable and frequently employed metrics in medical image analysis studies to evaluate the diagnostic performance of AI‐based CAD systems [75, 76]. As detailed in Table 7, for accuracy, fancy PCA improved mean performance by +3.00% with Adam and +2.93% with SGD, while for AUC, the mean performance enhancement was +2.66% with Adam and +2.76% with SGD and the fancy PCA approach yielded a consistent and statistically significant improvement in classification performance over conventional techniques (*p* < 0.05).

**TABLE 7:** Comparison of accuracy and AUC between models using fancy PCA and conventional augmentation, based on mean performance and paired *t*‐test results.

Metric	Compared methodologies	Mean performance (conventional)	Mean performance (fancy PCA)	Mean performance difference	Paired *t*‐test
Accuracy	Conventional vs. fancy PCA (Adam)	83.95%	86.96%	3.00%	*p* = 0.0021
	Conventional vs. fancy PCA (SGD)	83.07%	86.00%	2.93%	*p* = 0.0067
AUC	Conventional vs. fancy PCA (Adam)	90.60%	93.26%	2.66%	*p* = 0.0000
	Conventional vs. fancy PCA (SGD)	89.90%	92.67%	2.76%	*p* = 0.0000

One of the main limitations of this study was the relatively small and imbalanced dataset, which may lead to insufficient model training and introduce bias towards a specific class. To address potential bias, additional evaluation metrics beyond accuracy, including precision, sensitivity and F‐score, were computed and the results, shown in Tables 3 and 4, indicated that the proposed framework can maintain balanced performance and overcome bias [77, 78]. Moreover, ROC curves and the corresponding AUC scores were also employed as complementary and significant measures of diagnostic performance, which is particularly meaningful in diverse medical research topics, such as medical image analysis tasks, with widespread use in several studies to validate the efficacy of CAD systems in classifying medical images [76, 79, 80].

To mitigate the limitations of the dataset, fancy PCA could substantially enrich the diversity of the training set by increasing the size and improving class balance effectively and enabled the models to achieve desirable results despite the constraints of the original dataset. Primary limitations of photometric transformations, such as fancy PCA, include their dependence on variance in image intensities, which may result in unrealistic samples in overly strong transformations and the need for careful parameter tuning [81, 82]. These challenges were addressed through parameter calibration and evaluation across multiple architectures and optimisers, which ensured that the improvements obtained were both stable and generalizable.

In terms of generalisation, the proposed method was evaluated across a wide range of well‐established CNNs and optimisation algorithms, which consistently performed acceptably in classifying unseen test images. The superior mean classification accuracy and AUC values across these diverse settings, presented in Table 7, confirm that the proposed framework, which incorporates data augmentation via fancy PCA to enhance feature diversity and expand data variability, thereby supporting model generalisation, provides reliable and broadly applicable improvements rather than being limited to a specific model configuration [83, 84, 85].

Table 8 summarises proposed models in published recent papers and this study. Comparing the achieved results shows that the best‐performing model in this work can identify low and high‐grade meningiomas remarkably better on different MR images compared to other studies that have implemented different methods. Contrary to radiomic‐based models [31, 32, 33, 34, 35, 36, 38, 39, 40, 41, 43, 44], in addition to better performance, the proposed method in this work demanded neither complex preprocessing operations like ROI derivation nor hand‐engineering feature extraction methods, and it was expected to extract influential features from whole brain images automatically using convolutional and pooling layers instead of manual methods to detect meningiomas on MR images and make a distinction between low and high‐grade tumours without human intervention. The best‐attained model in this research could also achieve better classification results than other reviewed CNN‐based models [37, 40, 42, 45]. In [32, 36, 37, 40, 41, 44, 46], only one type of MR images was utilised to develop CAD systems, while a variety of MR types, that is, T1‐weighted, T2‐weighted, FLAIR and T1‐CE, were used in this work to train the models; thus they were able to detect low and high‐grade meningiomas on four different modalities of MR images. In comparison with prior studies, it is noteworthy that the proposed method achieved highly competitive and even superior performance outcomes, despite being trained and validated on a relatively limited dataset, which highlights both the robustness of the fancy PCA augmentation strategy and its potential applicability to larger‐scale datasets in future work.

**TABLE 8:** Summary of the best‐obtained models in related works and our proposed model.

Research	Data	No. of images/subjects	Data partitioning	Feature extraction	Classification	Best achievement
Chen et al. [43]	T1, T2, T1‐CE	609 subjects	Training: 307, Test: 238/64 subjects	Radiomics	LR	AUC: 91%
Duan et al. [44]	T1‐CE	340 subjects	Training: 70%, Validation: 30%	Radiomics	NB	AUC: 77.3%
Jun et al. [45]	T2, T1‐CE	318 subjects	Training: 257, Validation: 36, Test: 61 subjects	ResNet	FC	Acc: 72.1%, AUC: 77%
Hu et al. [38]	T1, T2, SWI, ADC	316 subjects	Nested LOOCV	Radiomics	RF	AUC: 81%
Ke et al. [35]	T1, T2, T1‐CE	263 subjects	Training: 184, Validation: 79 subjects	Radiomics	SVM	Acc: 89%, AUC: 91%
Duan et al. [41]	T1‐CE	188 subjects	Training: 70%, Test: 30%	Radiomics	SVM	Acc: 79%, AUC: 88.4%
Coroller et al. [32]	T1‐CE	175 subjects	Training: 131, Validation: 44 subjects	Radiomics	RF	AUC: 86%
Hamerla et al. [34]	T1, T2, FLAIR, ADC	138 subjects	10‐fold cross‐validation	Radiomics	XGBoost	AUC: 97%
Yang et al. [40]	T1	132 subjects	Train: 80%, Test: 20%	Radiomics, DL	LightGBM	Acc: 92.6%, AUC: 93.5%
Han et al. [39]	T2, T1 FLAIR, CE‐T1 FLAIR	131 subjects	Training: 80%, Test: 20%	Radiomics	SVM	AUC: 95.6%
Chu et al. [36]	T1‐CE	98 subjects	Training: 70%, Test: 30%	Radiomics	LR	Acc: 92.9%, AUC: 94.8%
Vassantachart et al. [42]	T1‐CE, FLAIR	96 subjects	Training: 76, Validation: 10, Test: 10 subjects	CNN	FC	Acc: 90%
Laukamp et al. [33]	T1, T2, FLAIR, T1‐CE, DWI, ADC	71 subjects	10‐fold cross‐validation	Radiomics	LR	AUC: 91%
Adil et al. [46]	FLAIR	38 subjects	Nested cross‐validation	Topological	XGBoost	AUC: 75%
Wodzinski et al. [37]	T1‐CE	174 images	4:1 split ratio for training and validation sets	CNN	FC	Acc: 74%
Yan et al. [31]	T1, T2, FLAIR, T1‐CE	131 images	10‐fold cross‐validation	Radiomics	SVM	Acc: 87%, AUC: 87%
Proposed model	T1, T2, FLAIR, T1‐CE	599 images/29 subjects	Training: 65%, Validation: 15%, Test: 25%	VGG‐19	FC	Acc: 98.90%, AUC: 99.89%

## 5 Conclusion

In conclusion, the intention was to examine the effectiveness of various CNNs with specific optimisers and hyperparameters alongside fancy PCA and conventional image augmentation techniques for automated meningioma grading. While the results demonstrated the promise of the proposed method, it is essential to acknowledge the limitations encountered during this research, which include the constraints of a small and imbalanced dataset, potential biases, the inherent limitations of photometric augmentation techniques like fancy PCA and the challenge of ensuring generalisation. Despite these limitations, the achieved results indicated that transfer learning combined with fancy PCA can become a worthy alternative to invasive methods and an outstanding AI assistant for clinicians to diagnose more accurately before treatment.

For further studies, it is proposed that models be trained using fine‐tuned CNNs trained with another enormous dataset, or train CNNs from scratch and compare the results with the best‐attained published ones. It is also recommended that FC layers be discarded and replaced with other classifiers to distinguish meningioma MR images into low and high grades based on extracted features and the difference between achieved classification accuracies be compared.

## Author Contributions

**Oktay Fasihi Shirehjini**: conceptualisation, formal analysis, software, validation, writing – original draft. **Farshid Babapour Mofrad**: conceptualisation, methodology, software, supervision, validation, writing – review and editing. **Mohammadreza Shahmohammadi**: conceptualisation, data curation. **Fatemeh Karami**: data curation.

## Conflicts of Interest

The authors declare no conflicts of interest.

## Data Availability

The data that support the findings of this study are available from the corresponding author upon reasonable request.
